# Development of high performance microwave absorption modified epoxy coatings based on nano-ferrites

**DOI:** 10.1038/s41598-024-55571-y

**Published:** 2024-03-02

**Authors:** W. M. Abd El-Gawad, E. M. Eldesouki, W. A. Abd El-Ghany

**Affiliations:** 1 Polymers and Pigments Department, National Research Centre, Dokki, Cairo Egypt; 2 Microwave Engineering Department, Electronics Research Institute (ERI), Cairo, Egypt; 3 Electron Microscope and Thin Films Department, National Research Centre, Dokki, Cairo Egypt

**Keywords:** Microwave absorption coatings, Nano-CuFe_2_O_4_, Nano-CaFe_2_O_4_, Dielectric properties, Magnetic properties, Reflection loss (RL), Chemical engineering, Inorganic chemistry, Chemical synthesis, Chemistry, Materials science, Nanoscience and technology, Physics

## Abstract

With the rapid spread of wireless technologies and increasing electromagnetic energy, electromagnetic waves (EMW) have become a severe threat to human health. Therefore, minimizing the harmful effects of electromagnetic wave radiation is possible through the development of high-efficiency EMW absorption coatings. The aim of this work was to generate microwave absorbance coatings containing synthesized nano-CuFe_2_O_4_ and nano-CaFe_2_O_4_. Firstly, nano-CuFe_2_O_4_ and nano-CaFe_2_O_4_ were synthesized using the sol–gel method. Then, their structure, electrical, dielectric, and magnetic properties were investigated to find out the possibility of using these materials in high-frequency applications (e.g., microwave absorbance coatings). After that, two dosages (2.5 wt% and 5 wt%) of nano-CuFe_2_O_4_ and nano-CaFe_2_O_4_ were incorporated into epoxy resin to prepare modified epoxy resin as microwave coatings. The dielectric studies show that the AC conductivity of the prepared samples is high at high frequencies. Additionally, the magnetic properties reveal a low coercivity value, making these samples suitable for high-frequency devices. The microwave results illustrate that adding nano-ferrites with high content enhances the absorption characteristics of the tested films. The results showed that the two films have two absorption bands with RL < –10 dB ranging from 10.61 to 10.97 GHz and from 10.25 to 11.2 GHz. The minimum return loss achieved for the two cases is −13 and −16 dB, respectively. Indicating that the film coated with CuFe has a better absorption value than the one coated with CaFe.

## Introduction

Recently, electromagnetic radiation has become widespread due to the rapid progress of wireless communication technology, particularly the explosive expansion of 5G and human over-reliance on many smart devices. As a result, the problem of electromagnetic pollution is getting more severe^1–5^. As a result, the problem of electromagnetic pollution is getting more severe. An excess of electromagnetic waves can cause electromagnetic disturbances, which can impede signal transmission and potentially disrupt the regular operation of electronic and intelligent infrastructure^6–9^. Even more concerning, studies show that prolonged exposure to high electromagnetic wave density is harmful to health and increases the threat of disease^1^. Most researchers agree that developing materials that absorb electromagnetic waves is a simple and effective way to minimise electromagnetic pollution.

Microwave absorption materials (MAMs) are useful materials capable of absorbing electromagnetic radiation and microwaves because of their low reflection and dispersion coefficients. The fundamental method of operation for microwave absorbers is to first transform microwave energy via a specific physical mechanism into a different kind of energy, and then to use a dissipation motion to transform that new type of energy back into heat energy. A high-performance microwave absorber must have a broad bandwidth and excellent absorption^10,11^. These materials are essential for a wide range of military and civil applications, such as healthcare, warfighter anti-radar monitoring, national defense security, and electronic reliability. The rapid development of information technology has led to a boom in the research of nanomaterials for microwave absorption applications in recent years. As a result, there has been a lot of interest in the development of high-performance MAMs at the nanoscale that are low-cost, have a broad bandwidth, and exhibit robust absorption^10^.

Ferrites are one of the most essential nanomertrais types that have attracted a lot of attention recently. Spinel ferrites are composite metal oxides with ferric ions that are crucial for industries. Their usual structural formula is MFe_2_O_4_, where M is a divalent metal ion like Zn^2+^, Co^2+^, Ni^2+^, Mg^2+^, Fe^2+^, Mn^2+^ etc., whose ionic radius varies from 0.6 to 1 Å^12,13^. Spinel ferrites have superior electric and magnetic properties, surface active sites, a large specific surface area, outstanding chemical stability, variable size and form, and varied applicability in every field of science and technology, among other distinctive physicochemical characteristics^14,15^. Moreover, high magnetic permeability, resistivity, excellent eddy current environmental friendliness, favorable mechanical properties, and magnetic loss are among the advantages of ferrite-based microwave absorbing materials, which are primarily employed to disperse electromagnetic radiation^16^.

On the other hand, radar-absorbing coatings with the benefits of easy production, high bonding force, and easy control of electromagnetic parameters are one of the most effective approaches to radar stealth technology to minimize the pollution of electromagnetic radiation^17–20^. It is well known that absorbing coatings are usually composed of MAM and an insulator matrix. MAMs are responsible for absorbing the EM wave, while the insulator matrix was to shield MAM and keep the shape of the radar-absorbing coatings^21,22^. Recently, epoxy coatings containing ferrites have attracted a lot of interest as potential microwave-absorption candidates with strong microwave-absorbing capabilities^16^. However, to the best of our knowledge, few works were pertaining to study the epoxy coatings containing ferrites as microwave absorbing coatings.

The aim of this work was to generate microwave absorbance coatings containing synthesized nano-CuFe_2_O_4_ and nano-CaFe_2_O_4_. After synthesis step, their structure, electrical, dielectric, and magnetic properties are investigated to find out the possibility of using these materials in high-frequency applications (e.g. microwave absorbance coatings). Then, two dosages (2.5 wt%, and 5 wt%) of nano-CuFe_2_O_4_ and nano-CaFe_2_O_4_ were incorporated into epoxy resin using ball-mill to prepare modified epoxy resin as microwave coatings. Thereafter, the return loss of four films coated with different contents of nano-ferrites is measured using VNA to check the microwave absorption of the proposed material. Also, the electromagnetic features of these samples are measured in terms of complex permittivity using Keysight Dielectric probe kit N1501A.

## Experimental part

### Materials

Calcium, copper, and ferric nitrates were obtained from WinLab, UK. Cetyltrimethylammonium bromide (CTAB) and sodium hydroxide were obtained from Adwic Co., Egypt. Epoxy resin consists of bisphenol A and polyamide as the curing agents. This ratio was obtained from Kemapoxy, Egypt. All the used extenders and solvents were supplied from local and international companies with normal chemical grades.

### Preparation of the prepared nano-ferrites

**Figure 1:**
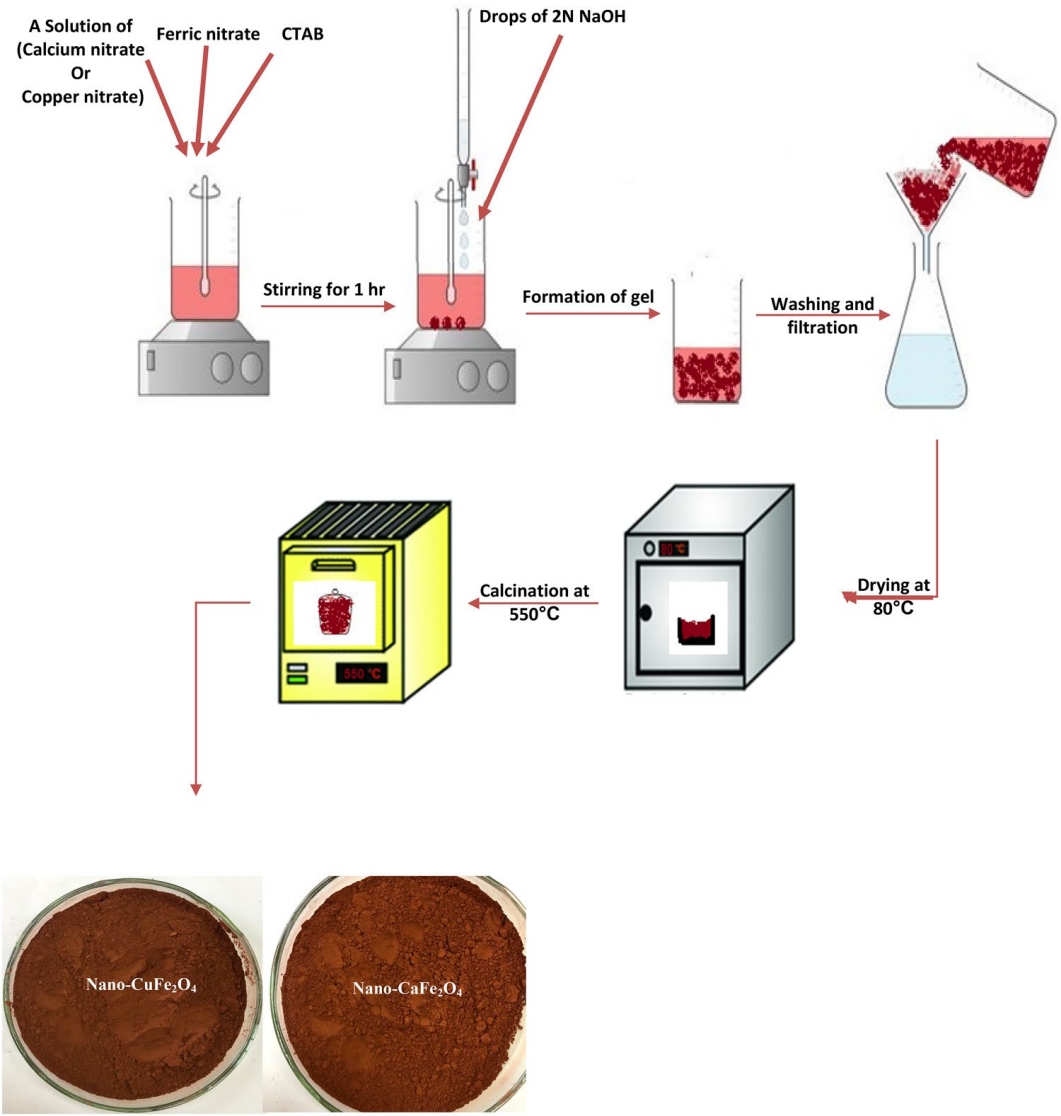
Synthesis process of both nano-ferrites.

As presented in Fig. 1, Fe(NO_3_)_3_⋅9H_2_O (0.2 M) and Ca(NO_3_)_3_⋅4H_2_O (0.1 M) were stirred at ambient temperature to prepare nano-CaFe_2_O_4_. Also, Fe(NO_3_)_3_⋅9H_2_O (0.2 M) and Cu(NO_3_)_3_⋅3H_2_O (0.1 M) were mixed to prepare nano-CaFe_2_O_4_. Then, 5% (w/v) CTAB was added to the two mixtures as a dispersing agent with continuous stirring at high speed. After that, 2 N NaOH was gradually added while being stirred frequently until the pH reached 8. Following thorough precipitation, the precipitates were cleaned with ethanol and deionized water before being set aside to dry at 80 °C. The dried dark red powders were then annealed at 550 °C in a muffle.

### Characterization of the prepared nano-ferrites

The morphology and elemental composition of the synthesized nano-ferrites were investigated using scanning electron microscope connected to energy dispersive X-ray spectroscopy (SEM/EDX-JEOL JED 2300). Transmission electron microscope (TEM-JEOL JX 1230) was used to determine the particle shapes and sizes of the nano-ferrites. X-ray powder diffraction patterns (XRD) were investigated at ambient temperature using a Philip’s diffractometer (Model PW1390), employing Ni-filtered Cu Kα radiation (λ = 1.5404 Å). The diffraction angle, 2θ, was scanned at a rate of 2°/min. Particle Sizing Systems\ZPW388 obtained from Santa Barbara, Calif., USA was used. X-ray photoelectron microscopy (XPS) was performed by (KRATOS-AXIS) Ultra spectrometer. The magnetic properties were performed at room temperature using a vibrating sample magnetometer (VSM; Lake Shore, USA), and the maximum magnetic field is 20 KG.

The dielectric and electrical properties of the nano-ferrites were carried out over a frequency range (10^–1^ to 10^7^) and at ambient temperature. A high-resolution alpha analyzer (Novocontrol Technologies, GmbH & Co. KG) is employed to achieve these measurements. For measurements, a disc of the sample is sandwiched between two gold-plated brass electrodes of 10 mm in diameter in parallel plate geometry. The complex dielectric function was obtained by:

1 $$\varepsilon *(\nu ) = \varepsilon ^{\prime}(\nu ) - i\varepsilon ^{\prime\prime}(\nu )$$

where *ε'* is a real part and *ε''* is an imaginary part or dielectric loss, *i* = *√−*1*,* and *ν* is the frequency. The complex conductivity σ* = *σ'* + *iσ"* and electric modulus M* = *M'* + *iM"* are determined and interpreted according to their relationships, as detailed in^23,24^. The frequency dependence of the three parameters (*ε′*, *M″*, and *σ′*) at room temperature will be considered here. These three parameters are interrelated to each other according to:

2 $$\varepsilon^{*} = \frac{1}{{M^{*} }} = \frac{{\sigma^{*} }}{{i\omega \varepsilon_{0} }}$$

which,

3 $$M^{{\prime\prime}} = \frac{{\varepsilon^{{\prime\prime}} }}{{\varepsilon^{{\prime}2} + \varepsilon^{{\prime\prime}2} }},\quad {\text{and}}\quad \sigma ^{\prime} = \varepsilon_{0} \omega \varepsilon^{{\prime\prime}}$$

### Production of coatings formulations containing nano-ferrites

In this work, a ball mill was used to prepare epoxy resin composite coating according to the proportion of nano-ferrites to epoxy resin mass fractions of 2.5% and 5%. Firstly, nano-CaFe_2_O_4_ or nano-CuFe_2_O_4_ was dispersed in a mixed solution of xylene and N-butanol (weight ratio of 7:3) using ultrasonic for 0.5 h. After that, the dispersed solution was mixed with the epoxy in a ball mill for 1 h. Finally, four paint formulations based on epoxy containing nano-CaFe_2_O_4_ or nano-CuFe_2_O_4_ at 2.5 and 5%, which were denoted as CaFe (2.5%), CaFe (5%), CuFe (2.5%), and CuFe (5%), were obtained.

### Methods of testing and evaluation of coating

Several ASTM standards, including hardness (ASTM D 6577), ductility (ASTM D 5638), impact resistance (ASTM D 2794), and pull-off strength (ASTM D 4541), were used to evaluate the coated films' elasticity, strength, and flexibility.

### Microwave setup

**Figure 2:**
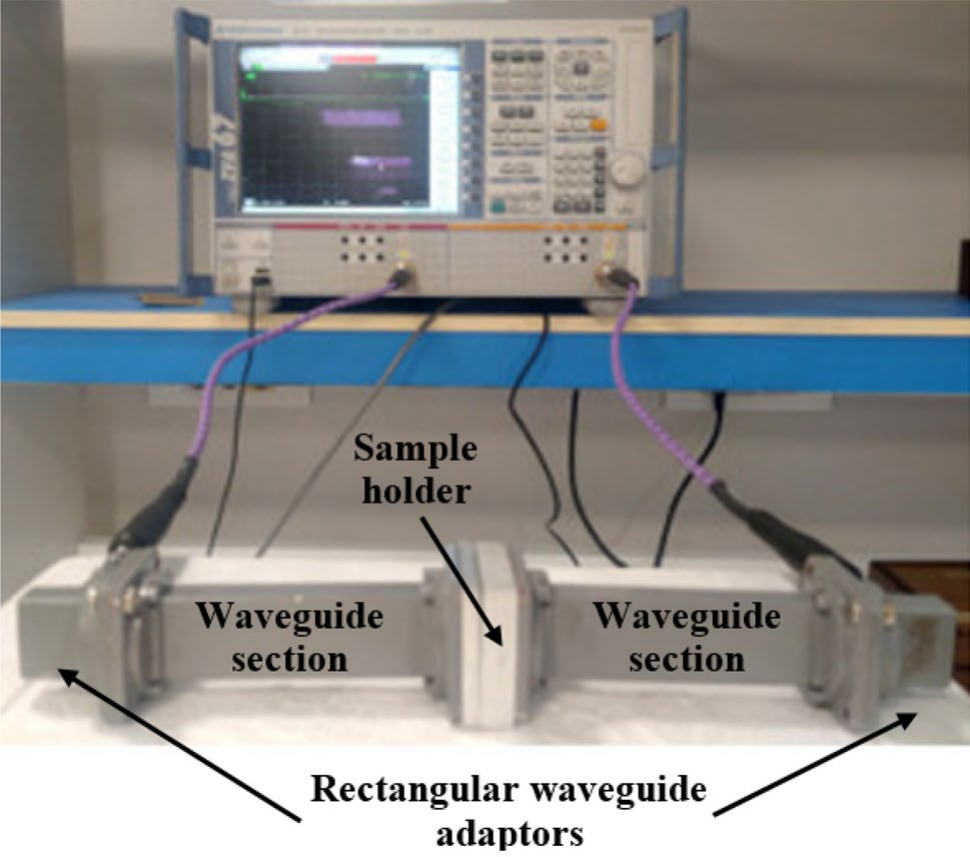
Measurement setup of the electromagnetic properties using TL technique.

$\tan \delta = {{\varepsilon ^{\prime}} \mathord{\left/ {\vphantom {{\varepsilon ^{\prime}} {\varepsilon ^{\prime\prime}}}} \right. \kern-0pt} {\varepsilon ^{\prime\prime}}}$ The ability of materials to absorb microwaves is directly linked to their electromagnetic characteristics. To investigate the absorption properties of microwaves, the electromagnetic features of coatings containing nano-CaFe_2_O_4_ and nano-CuFe_2_O_4_ were measured using SPEAG-DAK 3.5 (200 MHz to 20 GHz). In this work, one thickness (1.5 mm) was used, according to the literature^25^, to examine only the effect of both concentrations on the absorption properties of microwaves. These measurements included the samples' relative complex permittivity, Eq. (1), which is made up of real and imaginary parts. The real part represents the storage capacity of dielectric energy, while the imaginary part indicates the dissipation of dielectric. The dielectric loss tangent is used to describe the electromagnetic wave absorption properties of the absorbers. The reflection coefficients of rubber absorber sheets were measured using a transmission line technique in the 8–12 GHz frequency range at room temperature. A rectangular sample of rubber was placed in an aluminium specimen holder that connected the two waveguide sections, each 60 mm in length. The two waveguide sections were then connected to two ports of a Rhode and Schwartz model ZVA67 VNA vector network analyzer through two waveguide adaptors, as shown in Fig. 2. A full two-port transmission-reflection-line (TRL) calibration was performed to eliminate any loss due to the sample holder^9^. From the measured S_11_, the reflection loss (RL) was calculated as:

4 $$RL_{dB} = 20\log_{10} \left[ {S_{11} } \right]$$

## Results and discussion

### X-ray diffraction (XRD)

**Figure 3:**
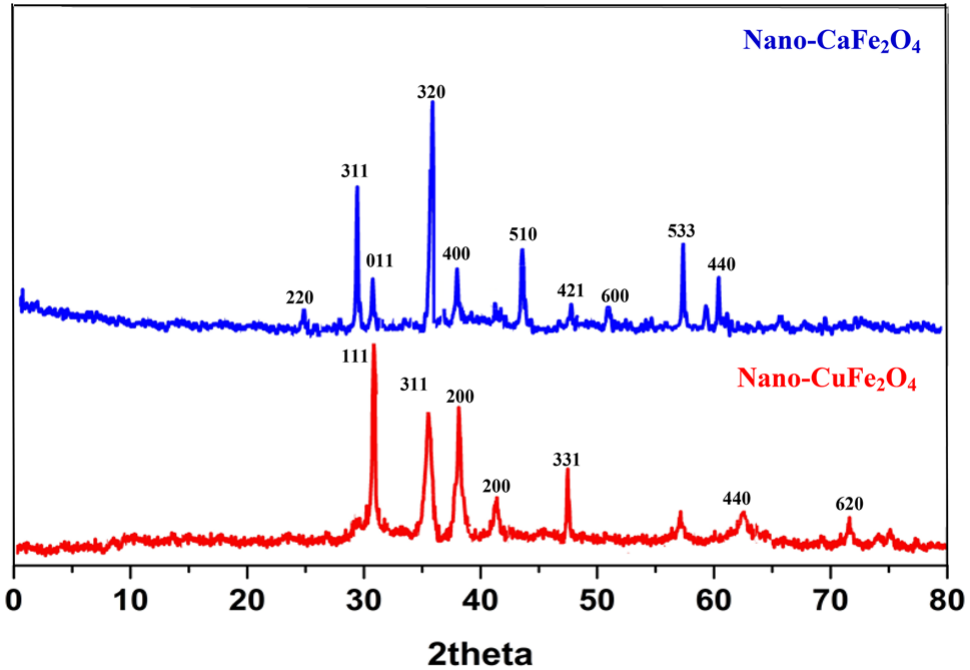
XRD of the synthesized nano-CaFe_2_O_4_ and nano-CuFe_2_O_4_. Transmission electron microscopy (TEM)/Scanning electron microscopy (SEM).

Figure 3 depicts the XRD patterns for nano-ferrites. According to the standard XRD data of nano-CaFe_2_O_4_ (JCPDS Card No. 78-4321), the distinctive diffraction peaks of nano-CaFe_2_O_4_ were observed at 2θ = 20°–70°^26^. The common nano-CuFe_2_O_4_ peaks are situated at 2θ = 30.1°, 35.6°, 38.7°, 42.8°, 47.3°, 57.1°, 62.95°, and 71.2°, and they correspond perfectly with the standard CuFe_2_O_4_ XRD data cart (JCPDS Card No. 034-0425)^27^. Furthermore, the Scherrer equation was used to examine the average crystal size of both nano-ferrites as follows:

$${\text{D = K}}\lambda {/}\beta {\text{cos}}\theta$$

where K = 0.89 is the Scherer constant, β is the width of the peak, θ is the Braggs diffraction angle, and λ is the wavelength of the X-ray^28^. The average crystal size of nano-CaFe_2_O_4_ was calculated to be 42.4 nm, while nano-CuFe_2_O_4_ was found to be 46.2 nm, clearly indicating that the synthesized nano-ferrites are of nano-scale.

**Figure 4:**
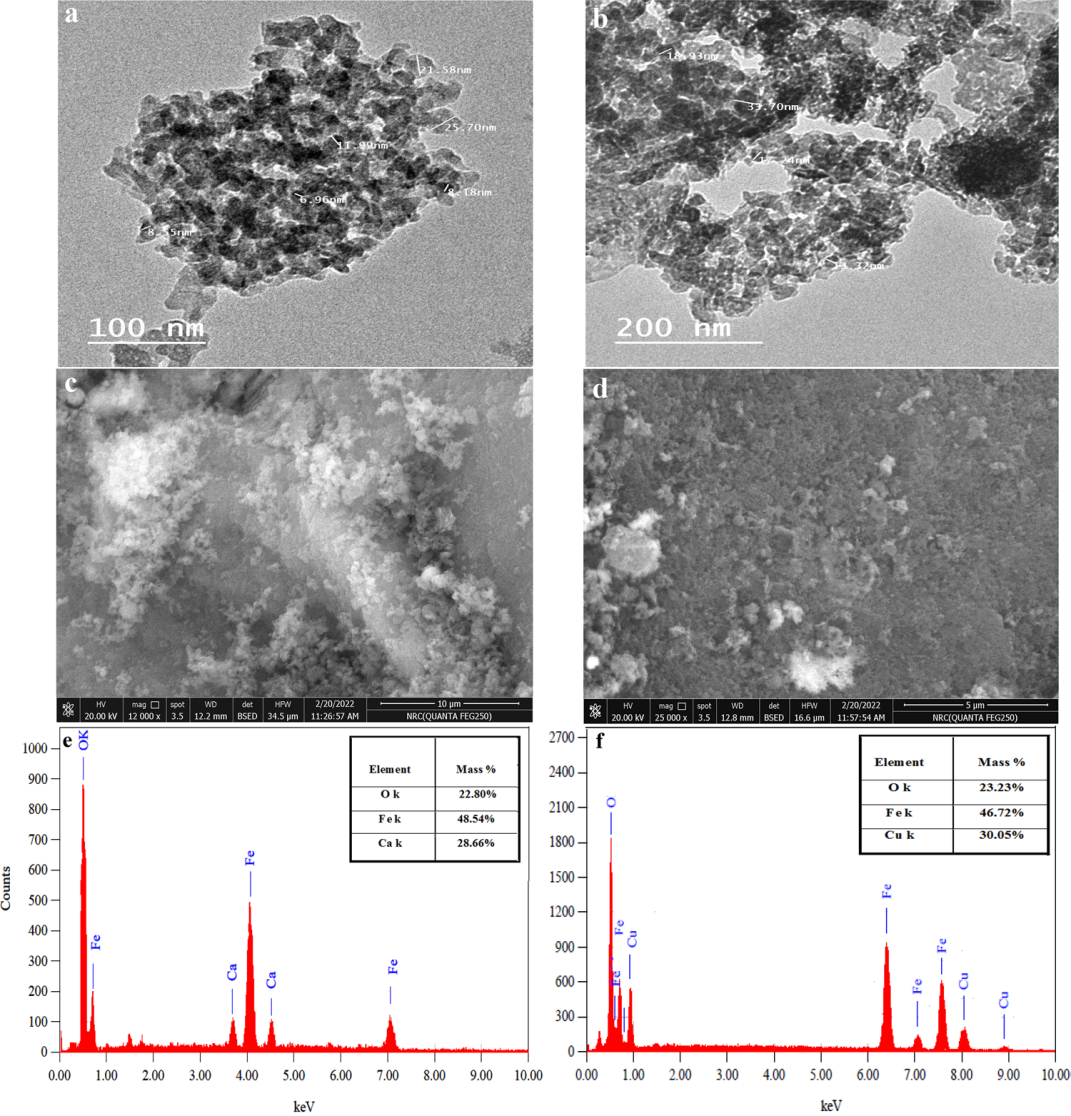
(**a,b**) TEM, (**c,d**) SEM, and (**e,f**) EDX analysis of the synthesized nano-CaFe_2_O_4_ and nano-CuFe_2_O_4_, respectively.

To examine the morphology and particle size of both nano-CaFe_2_O_4_ and nano-CuFe_2_O_4_, TEM and SEM were employed. TEM in Fig. 4a, b demonstrates that nano-CaFe_2_O_4_ has a particle size range of 8.18 to 25.7 nm and nano-CuFe_2_O_4_ has a particle size range of 13.37 to 33.70 nm. Additionally, the aggregation of nano-CaFe_2_O_4_ and nano-CuFe_2_O_4_ is made clear by TEM images, which may be triggered by the high surface energy and magnetic forces that occur between nanoparticles. On the other hand, SEM micrographs of synthesized nano-ferrites show their spinel structure, as seen in Fig. 4c, d.

### Energy dispersive X-ray analysis (EDX)

EDX analysis was employed to figure out the constituent elements of nano-CaFe_2_O_4_ and nano-CuFe_2_O_4_. According to an EDX investigation of the spectrum shown in Fig. 4e, f, the Fe, Ca, and O elements have been identified, which confirms the generation of nano-CaFe_2_O_4_. Additionally, EDX for nano-CuFe_2_O_4_ precisely determines the presence of Cu, Fe, and O.

### Zeta potential

**Figure 5:**
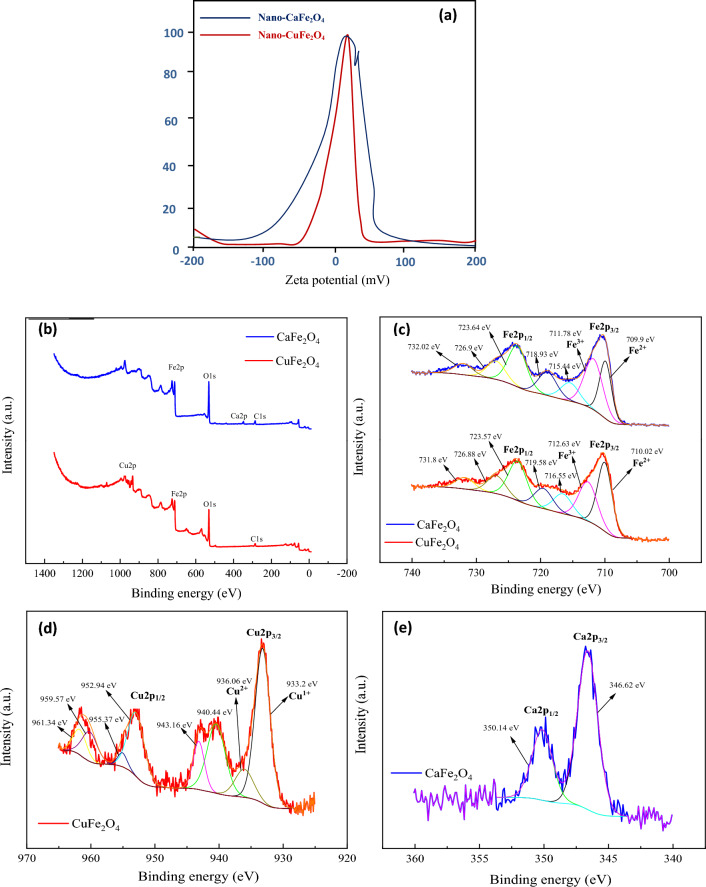
(**a**) Zeta potential of both nano-ferrites and their XPS analysis, (**b**) full XPS spectra, (**c**) Fe 2p spectra, (**d**) Cu 2p spectrum of nano-CuFe2O4, (**e**) Ca 2p spectrum of nano-CaFe2O4.

Figure 5 demonstrates the zeta potential of nano-CaFe_2_O_4_ and nano-CuFe_2_O_4_. Zeta potential is widely recognized for providing insight on the stability of nanoparticles in the media in which they are dispersed. The particle is considered stable if the potential is within the range of + 30 to −30 mV. The figure illustrates that both nano-ferrites fall within the indicated range, demonstrating that they are stable and do not aggregate under the intended usage conditions^29^.

### X-ray photoelectron spectroscopic (XPS) analysis

The possible valence states of each cation in nano-CuFe_2_O_4_ and nano-CaFe_2_O_4_ have been determined by performing XPS analysis. Figure 5b is the full XPS spectra of nano-CuFe_2_O_4_ and nano-CaFe_2_O_4_. The XPS spectra exhibit characteristic peaks at binding energies representing C 1s, O 1s, Fe 2p, Ca 2p, and Cu 2p. The Fe 2p_3/2_ and Cu 2p_3/2_ spectra can be well-fitted by two synthetic curves, indicating two possible valences for each ion, as shown in Fig. 5c, d. In Fig. 5c, two peaks are shown at binding energies of 710.02 and 709.9 eV for the Fe 2p3/2 line in nano-CuFe_2_O_4_ and nano-CaFe_2_O_4_, respectively. These peaks are attributed to Fe^2+^. On the other hand, the peaks at binding energies of 712.63 and 711.78 eV in both nano-CuFe_2_O_4_ and nano-CaFe_2_O_4_, respectively, are attributed to Fe^3+^^30,31^. Figure 5d displays two peaks at binding energies of 933.2 and 936.06 eV for the Cu 2p_3/2_ line, which are attributed to Cu^1+^ and Cu^2+^, respectively^30,32^. As shown in Fig. 5e, there are two peaks at 350.14 and 346.62 eV, corresponding to Ca 2p_1/2_ and Ca 2p_3/2_, respectively, demonstrating the presence of Ca^2+^^33–35^.

### Dielectric and electrical investigations

**Figure 6:**
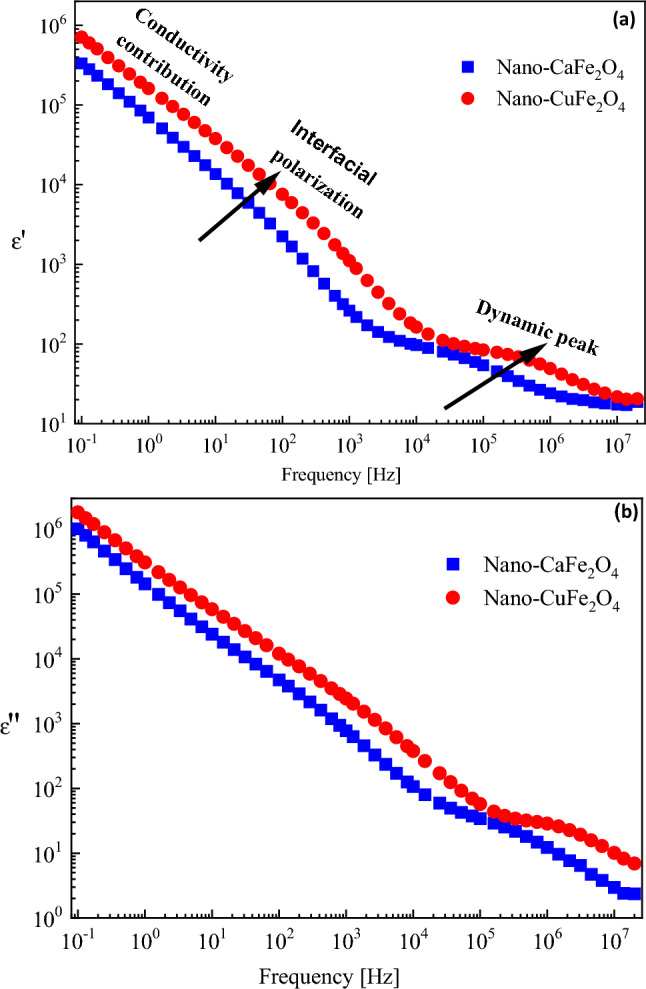
The frequency dependence of (**a**) permittivity, ε′and (**b**) dielectric loss, ε'′, for nano-CaFe_2_O_4_ and nano-CuFe_2_O_4_ under investigations at RT.

The real part of complex permittivity, *ε'*, is illustrated graphically as a function of frequency for CaFe_2_O_4_ and CuFe_2_O_4_ at room temperature in Fig. 6a. Three different trends are clearly shown in the figure. The lower range of frequencies (0.1–10 Hz) shows a clearly linear increase with decreasing frequency. This originated from the contribution of charge carriers’ transport, which causes ac conductivity. Further increases in frequency show shoulder-like behavior in the intermediate range of frequencies. This behavior may be attributed to the well-known interfacial polarization due to the accumulation of some ions at the interfaces at the borderers between different components and their heterogeneous structures^36^.

Similar behavior was also shown on the frequency dependence of dielectric loss *ε′′*(*f*)*,* as illustrated in Fig. 6b. This confirms that both parameters are not independent. At the higher limit of the frequency window (≥ 10 MHz), the permittivity values of both compositions collapse together and become independent of frequency. This can be explained according to the fact that the alteration of all kinds of polarizations lags behind the frequency of the externally applied electric field. This phenomenon became well known in many composites recently^37^.

**Figure 7:**
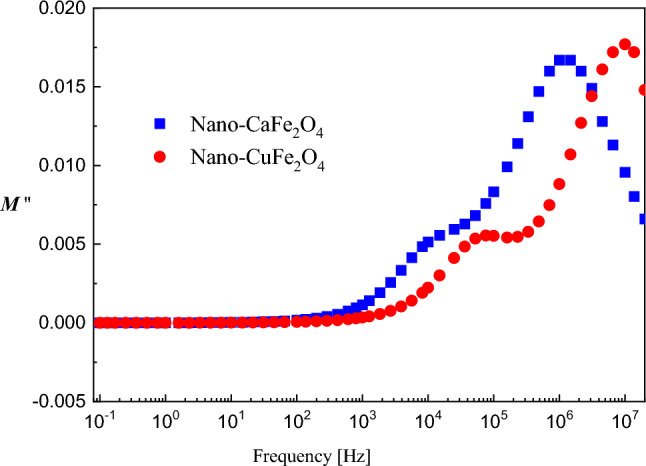
The frequency dependence of the imaginary part of electric modulus, M'', for the two samples under investigations at RT.

The representation of the imaginary part of the electric modulus *M''*(*f*) is usually used in dielectric characterization because it suppresses undesirable capacitance effects due to electrode contacts and provides a clear view of the DC conduction and dipole relaxation^36–38^. Figure 7 illustrates graphically the imaginary part of the electric modulus as a function of frequency. The two investigated processes are clear peaks in this representation. Both processes seem to be faster in the case of the CuFe_2_O_4_ composite. This may indicate that the degree of freedom of the Cu ions is higher than that of the calcium ions.

Generally, this investigated behavior can be explained on the basis of the Maxwell–Wagner bi-layered model, which is in agreement with Koop's dispersion phenomenological theory^39,40^. In this model, the dielectric material is composed of two layers. One of them is the grain boundary, which is a conductive layer, and the other is the grain boundary, which is a poorly conductive layer. The grain boundary is more active at lower frequencies, while its activity becomes less and the grain's activity dominates at higher frequencies. This is due to the movement of charge carriers (electrons) in the material that can move in the direction of the electric field by hopping mechanisms to the grain boundary at lower frequencies. The charge carriers accumulate at the grain boundary (a poorly conductive layer), forming space-charge polarization and causing the high value of the dielectric constant. As the frequency increases, the probability of reaching the grain boundary by the charge carriers is weak because they cannot follow the direction of the applied electric field, which thus causes the dielectric constant to decrease until it reaches a constant value^40–42^.

In the presented study, ε' has a high value at lower frequencies because the electron hopping between cations (Fe^3+^ ↔ Fe^2+^ in both the CaFe_2_O_4_ and CuFe_2_O_4_ samples besides Cu^2+^ ↔ Cu^1+^ in the CuFe_2_O_4_ sample as confirmed from XPS) takes place in the direction of the applied field, whereas it has a low value at higher frequencies due to the electron hopping between cations not following the alternating field^41,43^. Additionally, CuFe_2_O_4_ displayed improved dielectric properties compared to CaFe_2_O_4_ over the frequency range. This refers to the increase in charge carrier number that decreases the resistance of the sample, and consequently, the polarization increases, resulting in a high value of ε'^40,44,45^.

**Figure 8:**
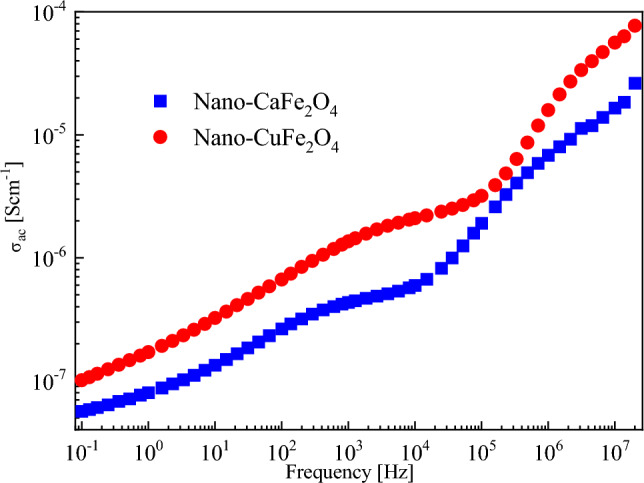
Frequency dependence of AC conductivity for CaFe_2_O_4_ and CuFe_2_O_4_ at room temperature.

The dependence of AC electrical conductivity on frequency for CaFe_2_O_4_ and CuFe_2_O_4_ at room temperature is shown in Fig. 8. It is observed that the conductivity increases as the frequency increases for the investigated samples due to the increased field applied to the charge carriers, which causes an increase in their hopping or tunnelling^46^. In ferrites, the electrical conductivity is due to the exchange of electrons between ions of the same element that have different valence states^41,47^. At low frequencies, the conductivity is low because of the grain boundary effect that reduces hopping electrons between Fe^3+^ and Fe^2+^. In contrast, the effect of the grain boundary becomes less at high frequencies, and the grain effect is dominant, and leading to hopping electrons^41^. Also, the AC conductivity of CuFe_2_O_4_ is higher than that of CaFe_2_O_4_ owing to the cationic distribution in the samples and the flow of charge carriers because of the grain effect, which is more active than the grain boundary effect^44,47^.

From the obtained results, it can be concluded that the real part of the complex permittivity and dielectric loss of the prepared nano-ferrites showed the dielectric behavior of these samples. Therefore, they can be used as dielectric materials in low-frequency devices. In addition, the AC conductivity of these samples has high values at high frequencies, making them also suitable for high-frequency devices^48^.

### Magnetic properties

**Figure 9:**
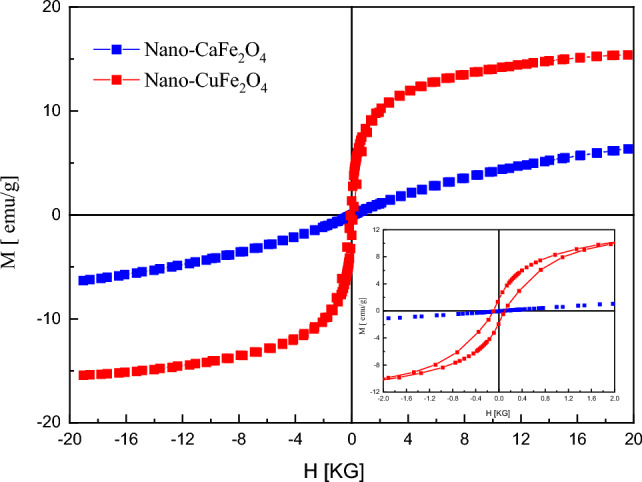
The magnetic hysteresis plots of nano-CaFe_2_O_4_ and nano-CuFe_2_O_4_ at room temperature.

Figure 9 shows the magnetic hysteresis plots of nano-CaFe_2_O_4_ and nano-CuFe_2_O_4_ at room temperature. The inset figure illustrates the magnified vision of the low-field magnetization region. The S shape in the hysteresis loop for the examined samples indicates the ferromagnetic behavior of the samples, which is somewhat strong for nano-CuFe_2_O_4_ while weak for nano-CaFe_2_O_4_. The values of magnetic parameters as coercivity, H_c_, remanent magnetization, retentivity, M_r_, saturation magnetization, M_s_, and squarence ratio, M_r_/M_s_, are calculated from hysteresis plots and tabulated in Table 1.

**Table 1:** Coercivity, H_c_, remanent magnetization, retentivity, M_r_, saturation magnetization, M_s_, and squarence ratio, M_r_/M_s_, of nano-CaFe_2_O_4_ and nano-CuFe_2_O_4_.

Sample	Coercivity H_c_ (G)	Remanent magntization (emu/g)	Retentivity M_r_ (emu/g)	Saturation magntization M_s_ (emu/g)	Squarence ratio M_r_/M_s_
CaFe_2_O_4_	23.135	0.0029777	0.015578	6.3349	0.00245908
CuFe_2_O_4_	99.364	1.7357	1.762	15.411	0.11433392

It is noticed that the value of M_s_ for nano-CuFe_2_O_4_ is higher than that of nano-CaFe_2_O_4_. The coercivity, Hc, of the samples is very low, which means that these samples can be considered soft magnets^49,50^. Moreover, the low value of coercivity indicates the negligible hysteresis loss of microwave energy which makes these samples candidates for high-frequency devices^2,51^.

### Coatings evaluation

#### Mechanical properties

**Figure 10:**
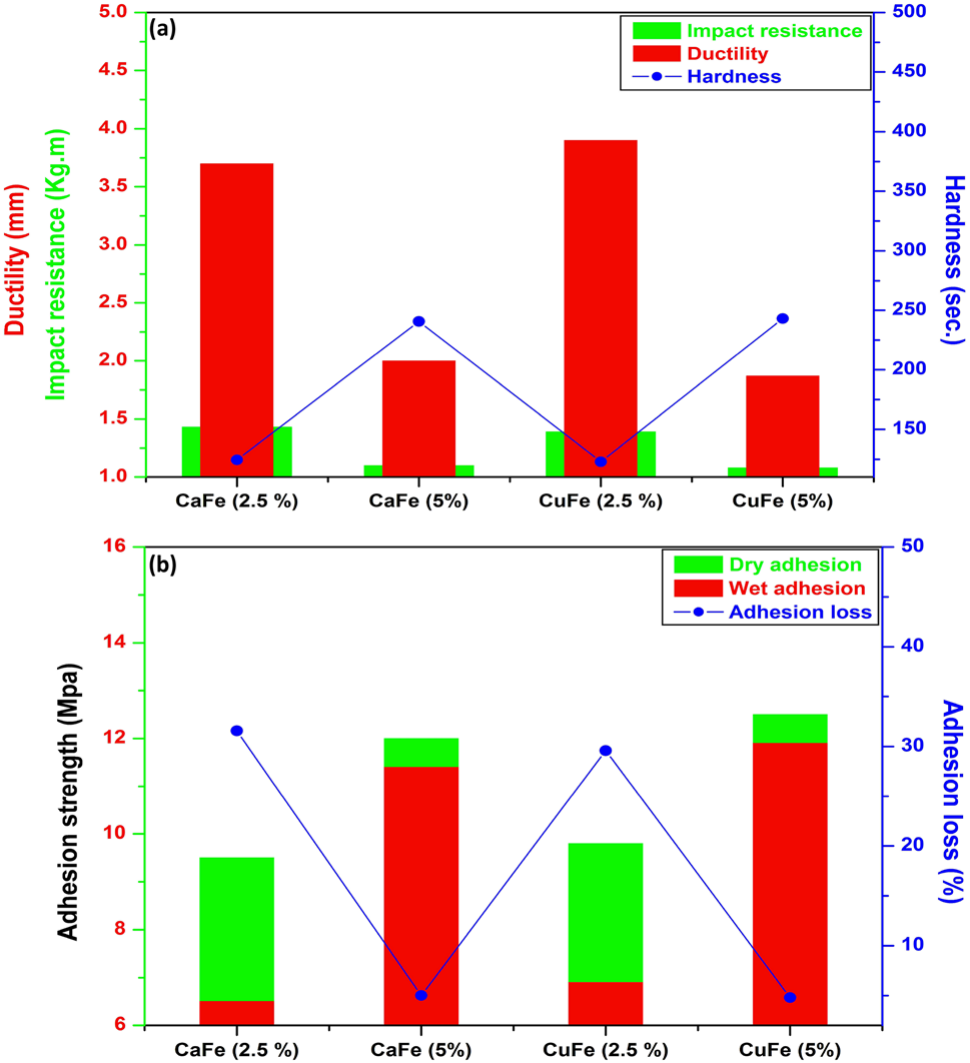
(**a**) Mechanical properties and (**b**) pull-off test results of the prepared coatings.

The mechanical features of CaFe (2.5%), CaFe (5%), CuFe (2.5%) and CuFe (5%) are shown in Fig. 10a. The figure shows that the hardness of CaFe (5%) and CuFe (5%) is higher than those containing 2.5%, which are approximately 240.56 and 243, respectively. Coatings containing 5% provided the best hardness values due to their high content in the film, resulting in the formation of a uniform film that had acceptable hardness. Meanwhile, the coatings with 5% had low impact resistance and ductility due to the presence of a significant quantity of epoxy rings that formed an inflexible and dense macromolecular framework. These characteristics, which result in a stiff and brittle structure, are a direct consequence of the hardener's depletion of all reactive sites.

#### Pull-off strength results

The pull-off test was used to determine the level of adhesion for coated panels that were both dry and wet. An equation has been used to estimate the adhesion loss (*ψ*) values as follows:

5 $$\psi = (\alpha_{D} - \alpha_{w} )/\alpha_{D} \times 100$$

where *α*_*D*_ denotes the dry adhesion and *α*_*w*_ represents the wet adhesion.

Figure 10b demonstrates that the films coated with CaFe (5%) and CuFe (5%) delivered the best dry and wet adhesion strengths with low adhesion loss, which are approximately 4 and 4.8%, respectively, while the adhesion loss of CaFe (2.5%) and CuFe (2.5%) is in the range of 31.57 and 29.59%, respectively. The presence of nano-ferrites with high content has improved both adhesion strength and reduced adhesion loss. This is attributed to the good arrangement of the nano-particles, which can block the whole voids in the matrix and form a tight film that could restrict the diffusion of aqueous solution through the film, so the adhesion is not affected^52^.

#### Microwave measurements

**Figure 11:**
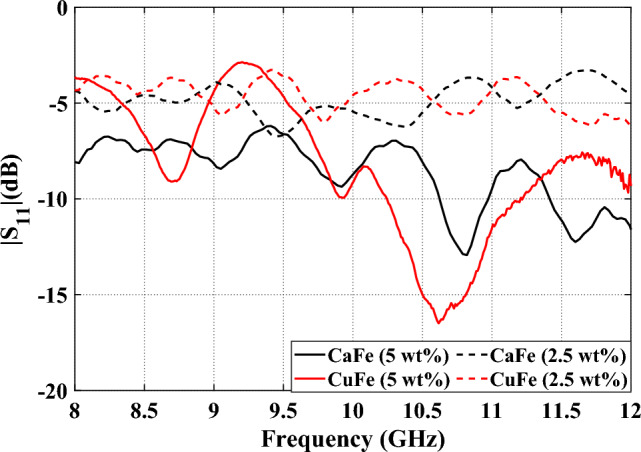
Frequency response of the reflection loss of the prepared coatings.

The variation of the return loss of the four films coated with CaFe (5% and 2.5%) and CuFe (5% and 2.5%) is shown in Fig. 11. From this result, it can be observed that adding nano-ferrites with high content enhances the absorption characteristics of the tested films, as the two films coated with 2.5% of nano-ferrites have a return loss of around −5 dB. However, the two films coated with (5% wt) of nano-ferrites have two absorption bands with RL < –10 dB ranged from 10.61 to 10.97 GHz for CuFe and from 10.25 to 11.2 GHz for CaFe, with centre frequencies of 10.75 GHz and 10.8 GHz for CuFe and CaFe, respectively, as shown in Fig. 13. The minimum return loss achieved for CaFe is −13 dB and for CuFe is −16 dB, respectively. Indicating that the film coated with CuFe has a better absorption value than that coated with CaFe, and the coated films containing a high ratio of both nano-ferrites (e.g., 5%) offer the best absorption value. Therefore, the dielectric measurements in the range 8–12 GHz have been performed for 5% of both CaFe and CuFe.

**Figure 12:**
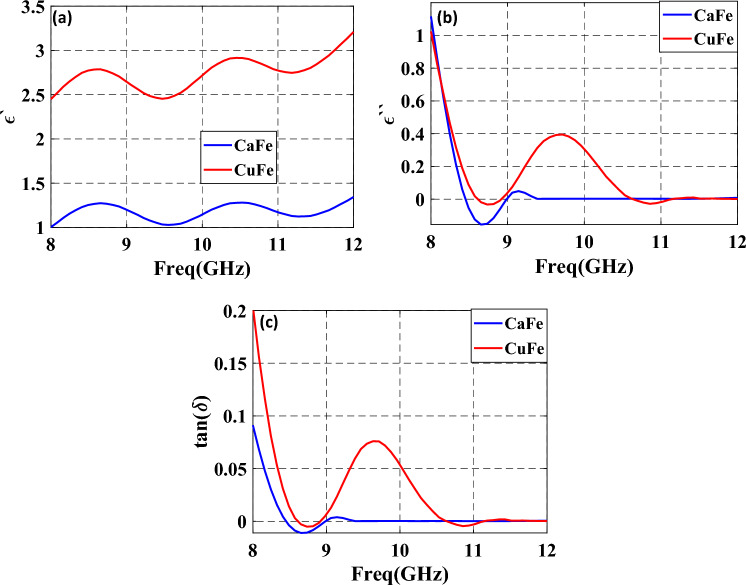
Frequency response of permittivity for two coated panels containing CaFe (5% wt) and CuFe (5% wt) as (**a**) real part, (**b**) imaginary part, and (**c**) dielectric loss tangent.

Figure 12 represents the measured $\varepsilon {^{\prime}}$, $\varepsilon {^{\prime}}{^{\prime}}$ and ${\text{tan}}{\delta }_{\epsilon }$ of the two films coated with CaFe and CuFe at a high concentration of 5%. From this figure, it can be observed that the values of $\varepsilon {^{\prime}}$ have a clear resonance at 10.75 GHz. Also, ${\text{tan}}{\delta }_{\epsilon }$ is an effective parameter for indicating the dielectric loss capability of the absorber. A moderate value of ${\text{tan}}{\delta }_{\epsilon }$ indicates a higher absorption rate. For ${\text{tan}}{\delta }_{\epsilon }\le 1$, good impedance matching will be obtained, indicating excellent microwave absorption performance. The measured ${\text{tan}}{\delta }_{\epsilon }$ obtained from the synthetic effect of $\varepsilon {^{\prime}}$ and $\varepsilon {^{\prime}}{^{\prime}}$ is indicated in Fig. 12c. It can be clearly seen from these two figures that the values of $\varepsilon {^{\prime}}{^{\prime}}$ and ${\text{tan}}{\delta }_{\epsilon }$ of the films fluctuate in the operating band of 8–12 GHz.

**Figure 13:**
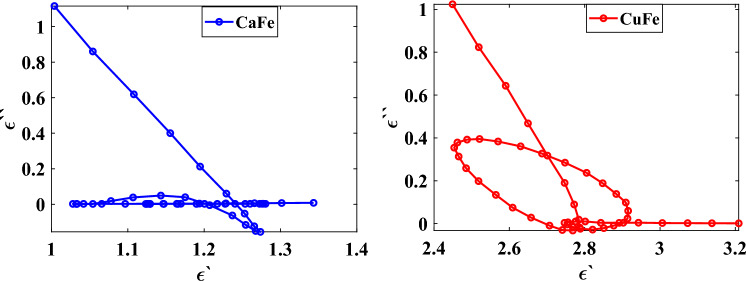
Typical Cole–Cole semicircles between the real part and the imaginary part of two coated panels of CaFe (5% wt) and CuFe (5% wt) in the frequency range of 8–12 GHz.

The relationship between $\varepsilon {^{\prime}}$ and $\varepsilon {^{\prime \prime}}$ of the two films of CaFe (5% wt) and CuFe (5% wt) is presented by the Cole–Cole plots in the frequency range 8 to 12 GHz, as shown in Fig. 13. It can be noted from these figures that the film coated with CuFe reveals a Debye relaxation process implied by a clear semicircle at the resonance frequency of 10.7 GHz, matching the resonance frequencies of the $\varepsilon {^{\prime \prime}}$ curve.

**Figure 14:**
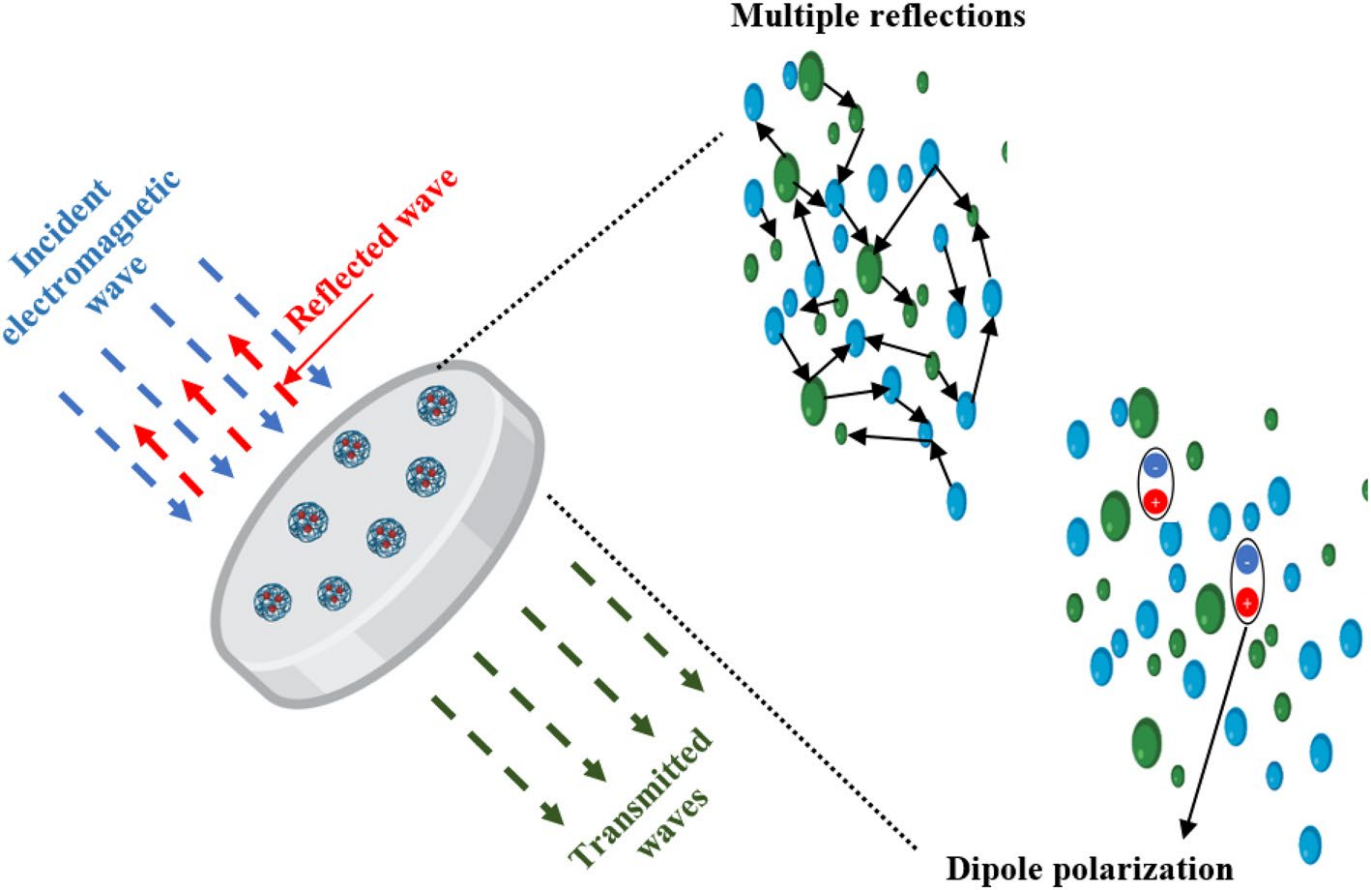
Schematic illustraion of microwave absorption mechanism of coatings containing nano-CaFe_2_O_4_ and nano-CuFe_2_O_4_.

The absorption mechanisms for the synthesized nano-CuFe_2_O_4_ and nano-CaFe_2_O_4_ can be explained based on their structural and electromagnetic properties. Nano-CuFe_2_O_4_ and nano-CaFe_2_O_4_ are both ferrite materials, which are known for their excellent electromagnetic properties and ability to absorb electromagnetic waves. These ferrites possess unique crystal structures that contribute to their absorption behavior. When exposed to microwave radiation, some electromagnetic microwaves transmit outside, and some waves interact with the nano-ferrite particles in the coatings. The absorption mechanism of both nano-CaFe_2_O_4_ and nano-CuFe_2_O_4_ involves a combination of dielectric loss, magnetic loss, multiple reflections, scattering, and interface effects. Figure 14 illustrates a schematic diagram of the mechanism of microwave absorption. The dielectric loss occurs because of the polarization of the electric dipoles within the material in response to the applied electric field of the electromagnetic wave. Both nano-CuFe_2_O_4_ and nano-CaFe_2_O_4_, exhibit dielectric properties that cause them to absorb and dissipate energy from the incident electromagnetic waves. Another absorption mechanism is magnetic loss. Nano-ferrites possess a high magnetic permeability, which allows them to interact strongly with the magnetic component of the electromagnetic wave. This interaction leads to the conversion of the magnetic energy into heat, resulting in energy absorption and attenuation of the electromagnetic wave. Also, the synthesized nano-ferrites contribute to multiple reflections and scattering of the incident electromagnetic waves. Due to their small size and unique morphology, the nano-ferrite particles can scatter the incoming waves in multiple directions. This scattering effect enhances the path length of the waves within the coatings, increasing the opportunity for absorption and dissipation of energy. The interfaces between the nano-ferrite particles and the surrounding matrix (such as epoxy resin) also play a role in the absorption mechanism. These interfaces can lead to additional absorption due to interfacial polarization and frictional losses. All these mechanisms collectively contribute to the effective absorption of microwave energy within the coatings, reducing the reflection of electromagnetic waves. It's important to note that the specific absorption characteristics of nano-CuFe_2_O_4_ and nano-CaFe_2_O_4_ can depend on factors such as their composition, particle size, morphology, and the surrounding matrix material^9^.

## Conclusions

In summary, new and cost-effective electromagnetic absorbing coatings that may effectively minimize electromagnetic radiation were successfully developed by the introduction of nano-CuFe_2_O_4_ and nano-CaFe_2_O_4_ modified epoxy resin. The study examined the mechanical characteristics of coatings that absorb electromagnetic waves, as well as the impact of coating impermeability and EMW absorption qualities. From this investigation, the following results are drawn:

- The addition of proper concentration of nano-CuFe_2_O_4_ and nano-CaFe_2_O_4_ can effectively improve the adhesion and hardness of epoxy resin coating. Among them, the optimized modified epoxy coating with nano-ferrites content of 5% exhibits the best enhancement effect, which can be attributed to the good arrangement of the nano-particles, which can block the whole voids in the matrix and form a tight film that could restrict the diffusion of aqueous solution through the film.
- The microwave results illustrate that adding nano-ferrites with high content (e.g., 5%) enhances the absorption characteristics of the tested films.
- From this result, it can be observed that adding nano-ferrites with high content enhances the absorption characteristics of the tested films, as the two films coated with 2.5% of nano-ferrites have a return loss of around −5 dB. However, the two films coated with (5% wt) of nano-ferrites have two absorption bands with RL < –10 dB ranged from 10.61 to 10.97 GHz for CuFe and from 10.25 to 11.2 GHz for CaFe, with centre frequencies of 10.75 GHz and 10.8 GHz for CuFe and CaFe, respectively. Indicating that the film coated with CuFe has a better absorption value than that coated with CaFe.
- Thus, the novel electromagnetic-absorbing coatings synthesized in this work can be widely applied for the purpose of protecting the environment from building electromagnetic pollution and reducing electromagnetic radiation caused by health hazards to people. They can also be used in television stations, airports, docks, and navigation beacons to eliminate reflection interference and reduce electromagnetic radiation.

## Data Availability

The datasets used and/or analysed during the current study available from the corresponding author on reasonable request. Figures 1 and 14 are prepared by the authors using normal tools.
